# State of the art in subtotal cholecystectomy: An overview

**DOI:** 10.3389/fsurg.2023.1142579

**Published:** 2023-04-21

**Authors:** Camilo Ramírez-Giraldo, Andrés Torres-Cuellar, Isabella Van-Londoño

**Affiliations:** ^1^ General Surgery Department, Hospital Universitario Mayor – Méderi, Bogotá, Colombia; ^2^ Escuela de Medicina y Ciencias de la Salud, Universidad del Rosario, Bogotá, Colombia

**Keywords:** difficult cholecystectomy, subtotal cholecystectomy, laparoscopic cholecystectomy, bile duct injury, critical view of safety

## Abstract

**Introduction:**

Subtotal cholecystectomy is a type of surgical bail-out procedure indicated when facing difficult laparoscopic cholecystectomy due to not reaching the critical view of safety, inadequate identification of the anatomical structures involved and/or risk of injury.

**Materials and methods:**

A comprehensive search on PubMed were performed using the following Mesh terms: Subtotal cholecystectomy and Partial cholecystectomy. The PubMed databases were used to search for English-language reports related to Subtotal cholecystectomy between January 1, 1987, the date of the first published laparoscopic cholecystectomy, through January 2023. 41 studies were included.

**Results:**

Subtotal cholecystectomy's incidence oscillates between 4.00% and 9.38%. Strasberg et al., divided subtotal cholecystectomies in “fenestrating” and “reconstituting” types based on if the remaining portion of the gallbladder was left open or closed. Subtotal cholecystectomy can sometimes be a challenging procedure and is associated to a high rate of complications such as biliary fistula, retained gallstones, subhepatic or subphrenic collections, among others.

**Conslusion:**

Subtotal cholecystectomy is a safe alternative when facing difficult cholecystectomy in which the critical view of safety is not reached in order to avoid complications. A classification system should be implemented in surgical descriptions to compare the different surgical techniques employed. In order to avoid bile leakage and cholecystitis of the remnant gallbladder, the surgical technique must be performed skillfully. There is still a current lack of information on alternative techniques such as omental plugging or falciform patch in order to judge their utility. There needs to be further research on long-term complications such as malignancy of the remnant gallbladder.

## Introduction

Subtotal cholecystectomy is a bail-out procedure undertaken when facing difficult laparoscopic cholecystectomy due to not reaching the critical view of safety, inadequate identification of the anatomical structures involved and/or risk of injury (1). Kehr (Germany) and Mayo (USA) were the first surgeons to perform a partial resection of the gallbladder during difficult cholecystectomy in the last decade of the nineteenth century. In Latin America it was first described by Alfonso Bonilla Naar (Bogotá, Colombia). From its first description to present time the literature on subtotal cholecystectomy has increased exponentially (2).

The 2020 World Journal of Emergency Surgery guide for acute calculous cholecystitis recommends performing subtotal cholecystectomy in situations where it's difficult to identify the anatomical structures needed or if there is a high risk of iatrogenic lesion (3). Possible bail-out procedures that can be performed during difficult cholecystectomy include intraoperative cholangiography, conversion to open procedure, aborting the procedure, or subtotal cholecystectomy. Subtotal cholecystectomy is considered the best bail-out technique when it's not possible to reach the critical view of safety during difficult cholecystectomy (1). The article “safe laparoscopic cholecystectomy: Adoption of universal culture of safety in cholecystectomy” by Gupta, et al. suggests subtotal cholecystectomy as a surgical bail-out procedure that permits safely finishing the procedure during difficult cholecystectomy (4).

Subtotal cholecystectomy is a procedure that has only become more relevant with the pass of time, with higher performance rates now more than ever. There isn't a current review of the literature that presents its definition, incidence, surgical technique and its variations, classification, and surgical outcomes in the short and long term. These aspects are key so the surgeon that decides to perform this procedure have evidence in the literature to back up when to employ the best surgical technique, know if cholecystectomy rates are within expected margins and to report the correct type of subtotal cholecystectomy employed so these can be compared with future research in mind.

Given what has been mentioned above, this study aims to perform an overview on subtotal cholecystectomy as a bail-out procedure in the face of difficult cholecystectomy.

## Materials and methods

A comprehensive search on Pubmed was performed using the following Mesh terms: “Subtotal cholecystectomy” AND “Partial cholecystectomy”. The PubMed database was used to search for English-language reports related to Subtotal cholecystectomy as shown in the following: (subtotal cholecystectomy) AND ((“1987/01/01”[Date - Create]: “2023/01/31”[Date - Create])) revealed 382 literature sources; (partial cholecystectomy) AND ((“1987/01/01”[Date - Create]: “2023/01/31”[Date - Create])) revealed 828 literature sources.

A total of 1,210 studies were identified. 41 studies were included; consisting of 2 meta-analyses, 5 systematic reviews, 1 national or international guidelines, 2 population-based studies, 22 observational (cross-sectional) studies, 3 case reports and 6 expert's opinion.

## Results

### Definition and prevalence

Subtotal cholecystectomy is a procedure which consists of removing portions of the gallbladder when it's not possible to identify the anatomical structures pertaining to the hepatocystic triangle during difficult cholecystectomy. In 2015 Strasberg, et al., recommended that the term “subtotal cholecystectomy” should be used in place of all possible terms related to partial cholecystectomy (5).

Subtotal cholecystectomy's proportion oscillates between 4.00% and 9.38% (6–10) according to the different sources reviewed. It has been employed as a safe alternative during laparoscopic cholecystectomy when the critical view of safety is not reached. Due to this same reason, there has been a lower rate of conversion to open cholecystectomy; moreover, newer generation surgeons seem to have a lower degree of expertise when performing open cholecystectomy and as a result prefer performing subtotal laparoscopic cholecystectomy over the former (11). In a national sample including 290,855 patients between 2003 and 2014 an increase in subtotal laparoscopic cholecystectomy proportion was seen from 0.10% to 0.52% with a decrease in conversion to open cholecystectomy proportion from 10.5% to 7.6% (12).

### Risk factors

Cholecystectomy difficulty can be predicted in a preoperative manner by using clinical, radiologic and laboratory findings, however, it becomes apparent only intraoperatively (13). The amount of preoperative factors for difficult cholecystectomy are directly proportional to the probability of performing subtotal cholecystectomy (14). Because subtotal cholecystectomy is one of the treatment options for difficult cholecystectomy, we can thus assume that factors associated to difficult cholecystectomy are similar to those associated with subtotal cholecystectomy.

Multiple factors associated to subtotal cholecystectomy have been identified such as male sex (OR = 2.59) (7), older age (OR = 1.23) (7), ASA score of ≥3 (OR = 3.84) (15), white blood cells (WBC) (OR = 2.02) (7), albumin level (OR = 0.31) (16), preoperative diagnosis of acute on chronic cholecystitis (OR = 5.47) (7), acute cholecystitis (OR = 2.69) (16), higher Tokyo grade for severity of acute cholecystitis (OR = 2.37) (17), history of liver disease (OR = 8.40) (16), time from onset to surgery (OR = 5.31) (15), previous biliary tract drainage (cholecystostomy) (OR = 2.66) (16, 18). Image findings have also been reported such as obscuration of the gallbladder wall around the neck (OR = 10.56) (15) and disruption of the common hepatic duct (OR = 3.92) (15), amongst others (Table 1).

**Table 1:** Risk factors associated with subtotal cholecystectomy.

Author	Year	Factor	OR	CI 95%
Annie Tang et al. (7)	2020	Attending experience (≤5 vs. >5 años)	0.66	0.41–1.06
		Older age	1.23	1.02–1.49
		Male sex	2.59	1.53–4.40
		White blood cells above 10.3	2.02	1.15–3.55
		Acute on chronic cholecystitis	5.47	1.28–23.43
Atsushi Kohga et al. (15)	2021	ASA score of ≥3	3.84	1.46–11.11
		Time from onset to surgery (on or after 9 days)	5.31	2.07–13.61
		Thickness of GB wall around the neck (≥3 mm)	10.56	3.16–35.21
		Disruption of common hepatic duct	3.92	1.11–13.83
Mitsugi Shimoda et al. (16)	2021	Previous biliary tract drainage (cholecystostomy)	2.66	1.08–6.56
		Acute cholecystitis	2.69	1.09–6.65
		Albumin level	0.31	0.09–0.96
Alexandria Byskosh et al. (17)	2023	Age	1.03	0.98–1.08
		Female	0.44	0.17–1.10
		History of liver disease	8.40	1.24–51.53
		Preoperative white blood cell count	1.10	1.00–1.21
		Time from admission to surgery	1.00	0.98–1.01
		Higher Tokyo grade for severity of acute cholecystitis	2.37	1.06–5.33

### Classification

**Figure 1:**
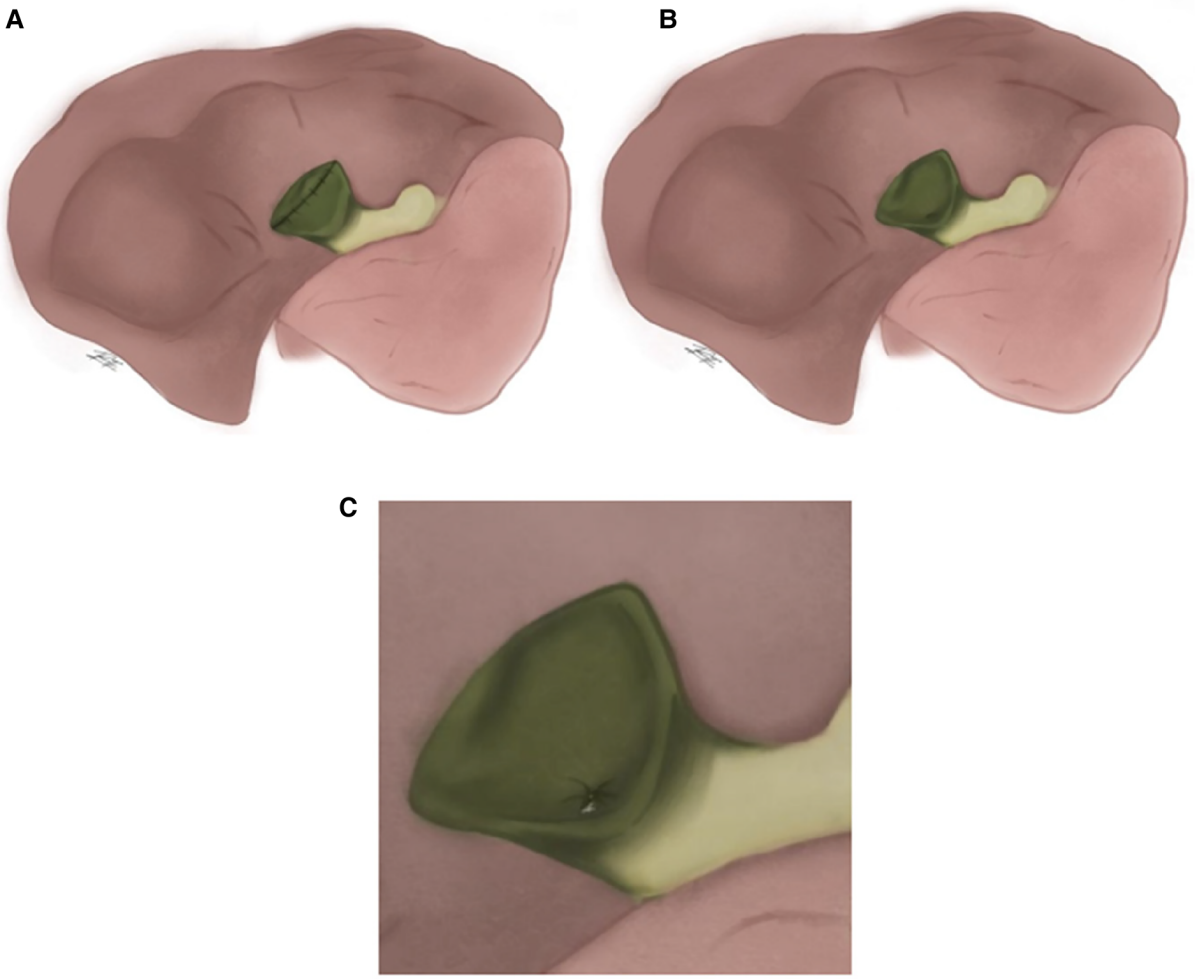
(**A**) Reconstituting type. (**B**) Fenestrating type. (**C**) Fenestrating type with internal suture-closure of the cystic duct.

Strasberg, et al., divided subtotal cholecystectomies in “fenestrating” and “reconstituting” types based on if the remaining portion of the gallbladder was left open or closed (Figure 1). Fenestrating subtotal cholecystectomy can be performed with or without internal suture-closure of the cystic duct. In both types, the gallbladder portion in contact with the liver can be left as is or resected. “Reconstituting” type cholecystectomies are performed in an attempt to avoid biliary fistulas, however they pose a higher risk of new gallstone formation in the remnant gallbladder and are consequently at higher risk of recurrent biliary disease (5).

**Figure 2:**
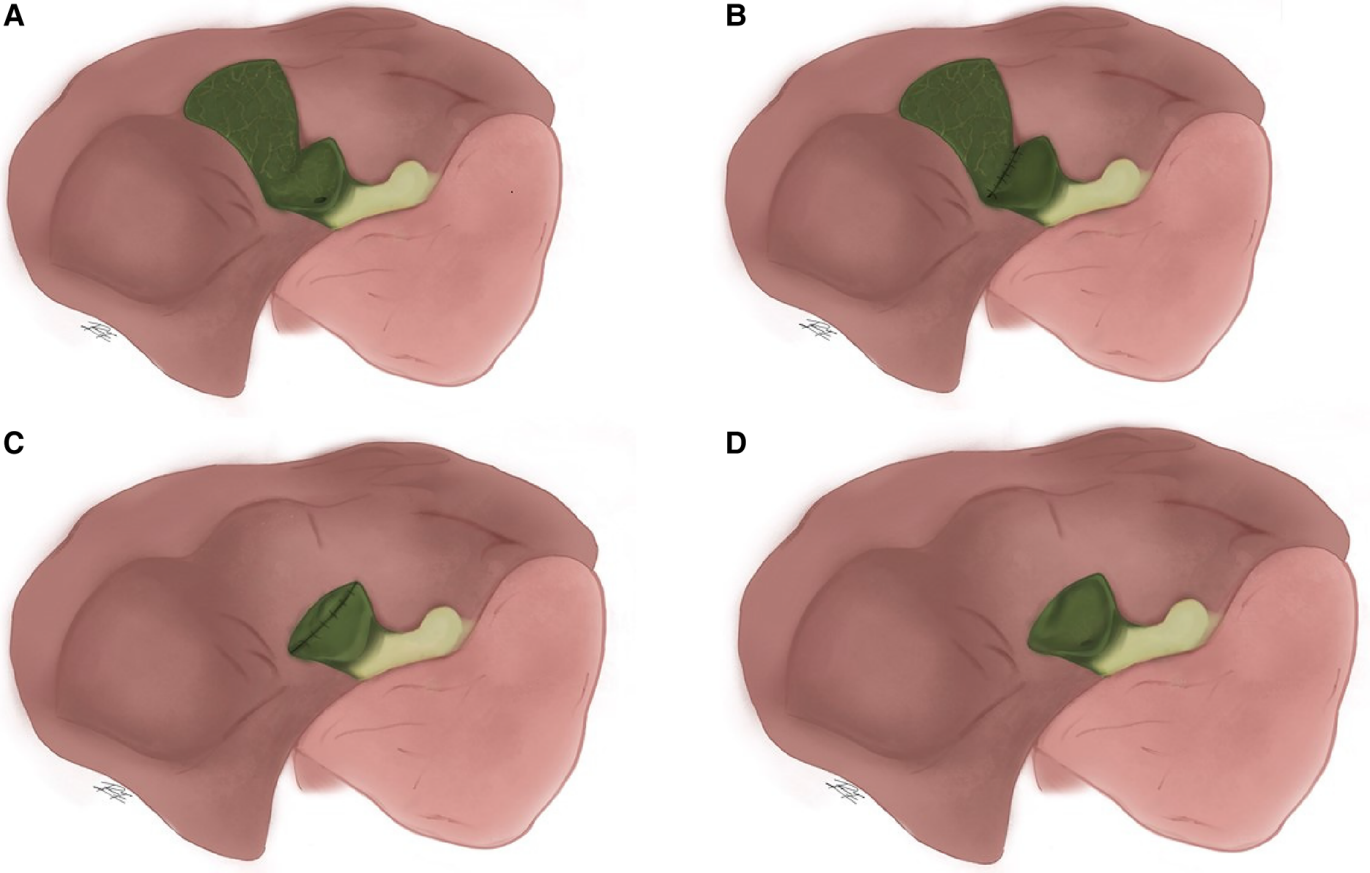
Henneman's method of subtotal cholecystectomy. (**A**) Leaving the posterior gallbladder wall attached to the liver and the remainder of the gallbladder stump open; (**B**) Like type A but with the gallbladder stump closed; (**C**) Resection of both the anterior and posterior gallbladder walls with the stump closed; and (**D**) Like C but with the gallbladder stump open.

A more complete classification has been proposed by Henneman in which subtotal cholecystectomy is divided into 4 methods: (A) Leaving the posterior gallbladder wall attached to the liver and the remainder of the gallbladder stump open, placing a drain; (B) Like type A but with the gallbladder stump closed, with or without drain placement; (C) Resection of both the anterior and posterior gallbladder walls with the stump closed, without drain placement; and (D) Like C but with the gallbladder stump open, placing a drain (Figure 2) (8, 19).

**Figure 3:**
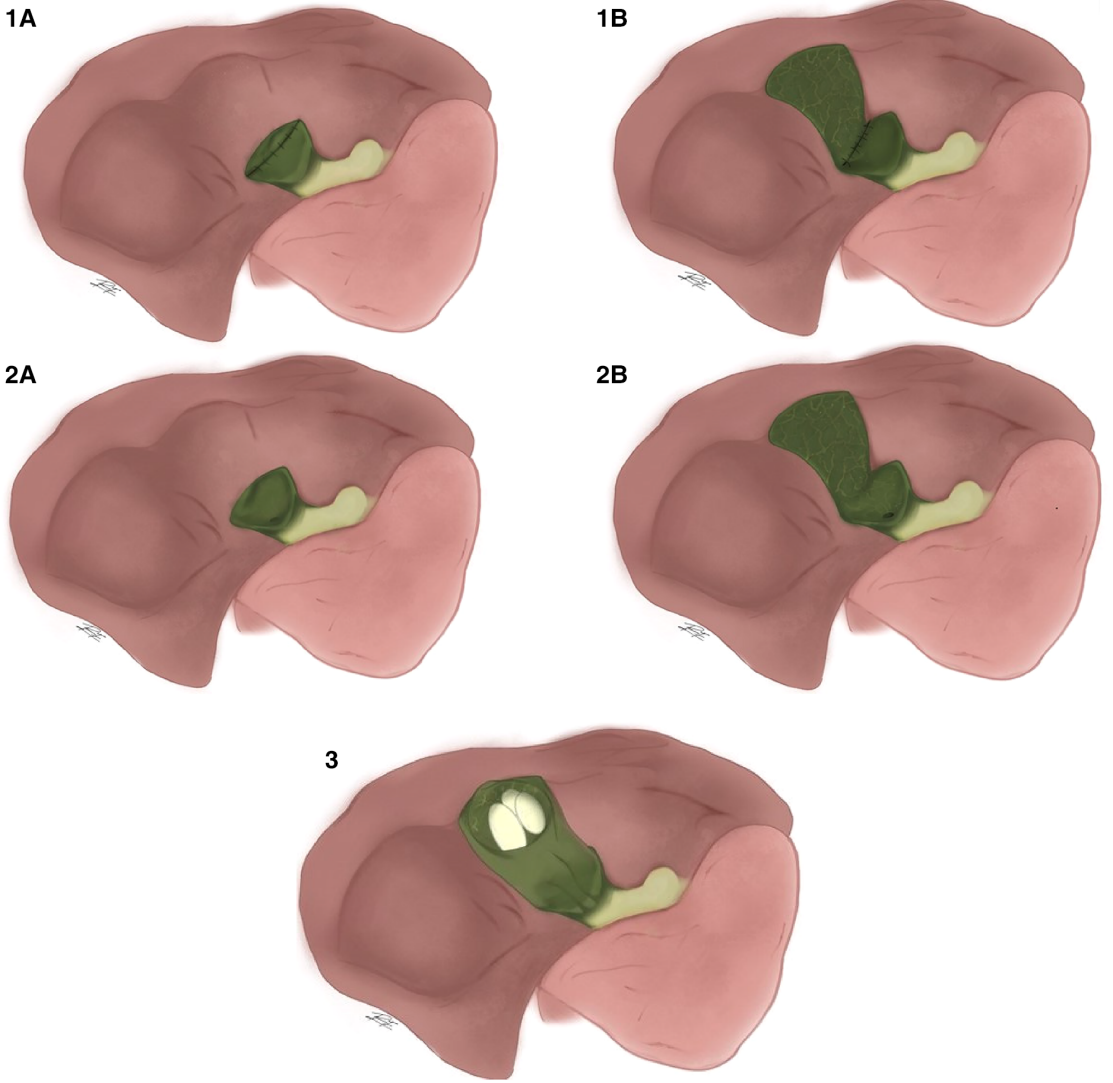
Purzner's classification of subtotal cholecystectomy. (**1A**) Complete dissection of the posterior gallbladder wall with the Hartmann's pouch closed; (**1B**). Preservation of the posterior gallbladder wall with the Hartmann's pouch closed; (**2A**) Full mobilization of the posterior gallbladder wall off the liver bed with the remnant gallbladder open; (**2B**) Preservation of the posterior gallbladder wall but with the remnant open and (**3**) For cases with extensive adhesions and inflammation, the gallbladder in fenestrated high up on the fundus and gallstones are evacuated while leaving the remnant gallbladder open.

Over time this classification has been modified. Purzner et al., establishes 5 subtypes of subtotal cholecystectomy depending on if there is or isn't closure of the gallbladder stump, if the gallbladder portion attached to the liver is resected or left as is and lastly into subtype 3 for cases with extensive adhesions and inflammation, the gallbladder is fenestrated high up on the fundus and gallstones are evacuated while leaving the remnant gallbladder open. This type also refers to damage-control cholecystectomy, used only when hostile adhesions prevent exposure of the gallbladder altogether (Figure 3) (20).

**Figure 4:**
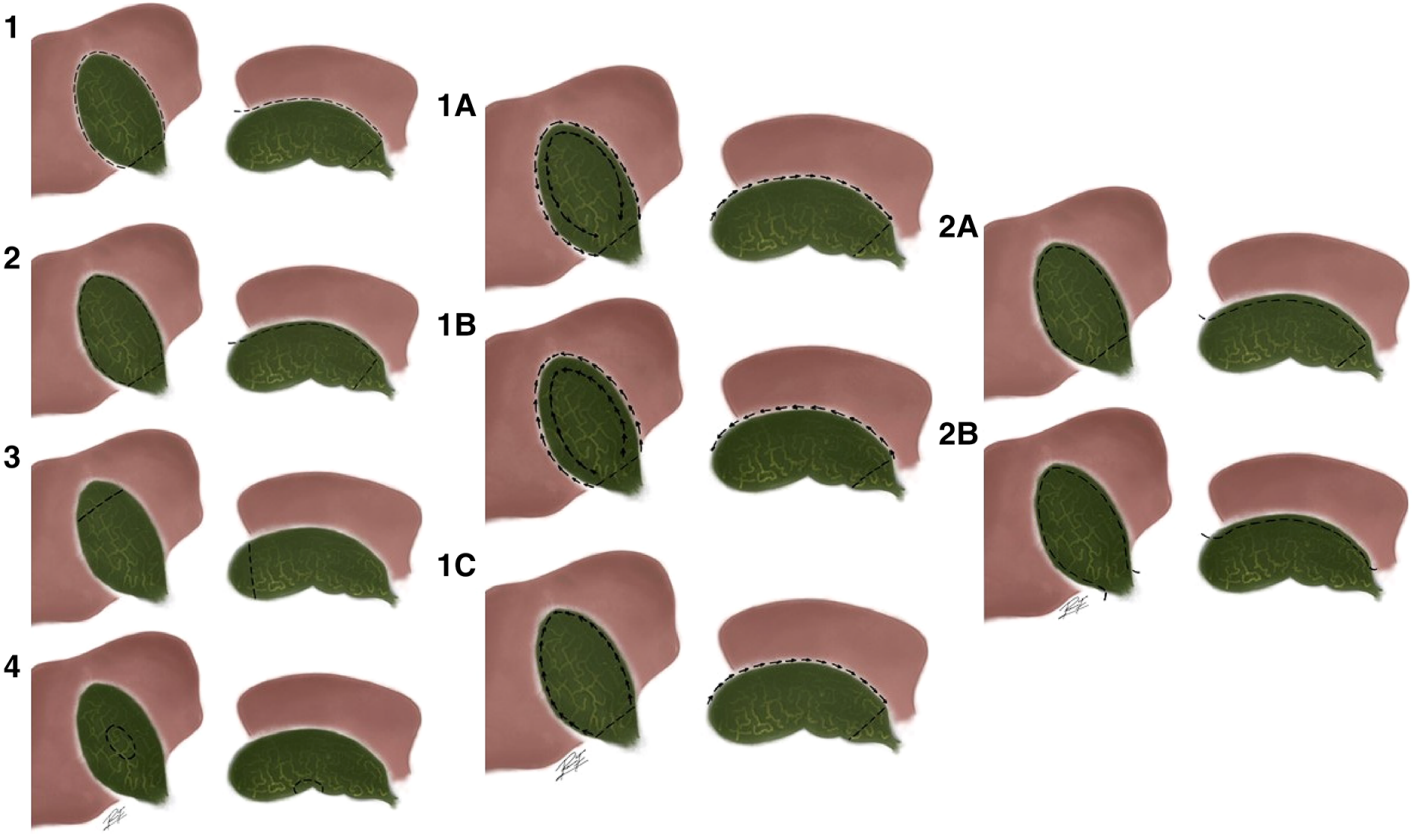
Lunevicius’ classification subtotal cholecystectomy. (**1**) A circular excision of a considerable portion of the gallbladder; (**2**) A longitudinal removal of a considerable portion of the visceral wall of the gallbladder; (**3)** Fundectomy (in case of a solid inflammatory mass around the body and neck of the gallbladder in a high-risk patient); (**4**) Wedge resection of the gallbladder (possible variant, if the gallbladder is true intrahepatic). Type 1 can then be divided into 3 more subtypes: (**1A**) Fundus-down first, circular transection of the proximal portion of the gallbladder second; (**1B**) Transection of proximal portion of the gallbladder first, body-fundus-up second; (**1C**) Visceral wall first, hepatic wall second. And then type 2 can be divided into 2 more subtypes: (**2A**) Excision of the visceral wall, just; (**2B**) Transection of the cystic duct, excision of the visceral wall (and a part of the hepatic wall) of the gallbladder.

Lunevicius describes a type of classification different to previous ones. Other, previous classifications aimed to subdivide the type of closure on subtotal cholecystectomy; on the other side, this classification hoped to distinguish on the different possible variants of gallbladder resection (Figure 4) (21).

Lastly, Lunevicius recently proposed replacing the term “fenestrating” and “reconstituting” subtotal cholecystectomy for subtotal open-tract and subtotal closed-tract cholecystectomy, considering that the terms “fenestration” and “reconstitution” lack specificity (2).

### Surgical technique

#### Subtotal fenestrating cholecystectomy

The technique consists in opening the free wall of the gallbladder from the fundus downwards or from the body upwards using cauterization; trying to remove as much of the free side of the wall as possible while staying above the safety line between Rouviere's sulcus and the umbilical fissure thus avoiding dissection of this area of the hepatocystic triangle, being careful not to dissect it off the cystic plate. When resecting the gallbladder walls the cystic artery should be clipped though in multiple cases it may be thrombosed due to severe inflammation. All gallstones must be retrieved until the gallbladder's infundibulum is reached ensuring every stone has been removed. If the internal orifice can be clearly seen it can be sutured however in most cases due to severe inflammation this is not feasible. The remaining portion of the gallbladder attached to the cystic plate should be ablated with electrocautery, this may prevent remnant mucosal secretion and cause inflammatory adhesions leading to earlier closure of the cystic duct (13).

#### Subtotal reconstituting cholecystectomy

The difference with the “fenestrating” type consists in closing the remaining gallbladder stump, which can be done with either stapled or manual sutures. When closing the remnant gallbladder it's necessary for the remaining portion to be slightly bigger than accustomed for the “fenestrating” type (5).

#### Subtotal fenestrating cholecystectomy with falciform patch

**Figure 5:**
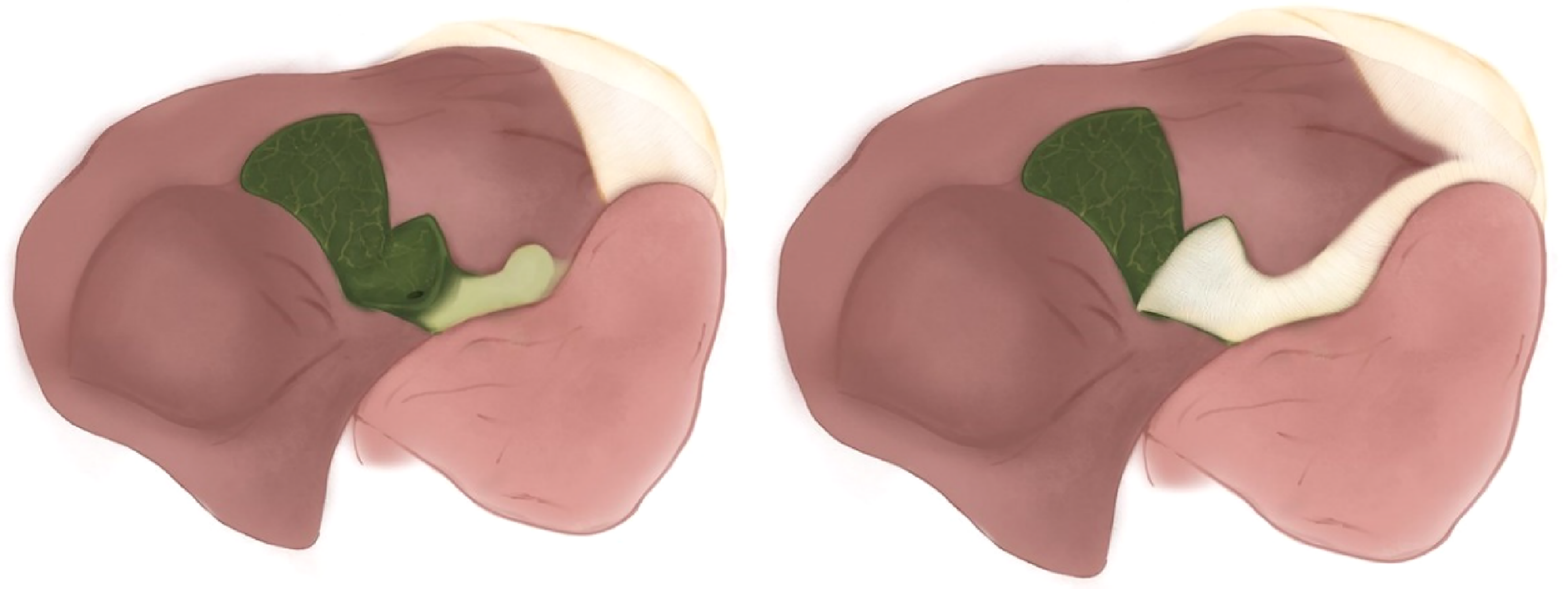
Falciform ligament patch.

A fenestrating subtotal cholecystectomy is performed as previously described. The posterior gallbladder wall can be removed or left as is and all gallstones must be removed. The patch is made by dividing the round ligament and falciform ligament as close as possible to the umbilicus going cephalically towards the liver staying sub-centimeter away from the abdominal wall, thus securing a vascularized pedicle flap. The pedicle should be long enough to reach the gallbladder remnant without tension. The edges of the falciform ligament are then clipped or sutured to the remnant gallbladder staying away from the hepatocystic triangle. A closed drainage system should be placed near the patch to avoid bile leak (Figure 5) (22).

#### Subtotal fenestrating cholecystectomy with omental plugging

**Figure 6:**
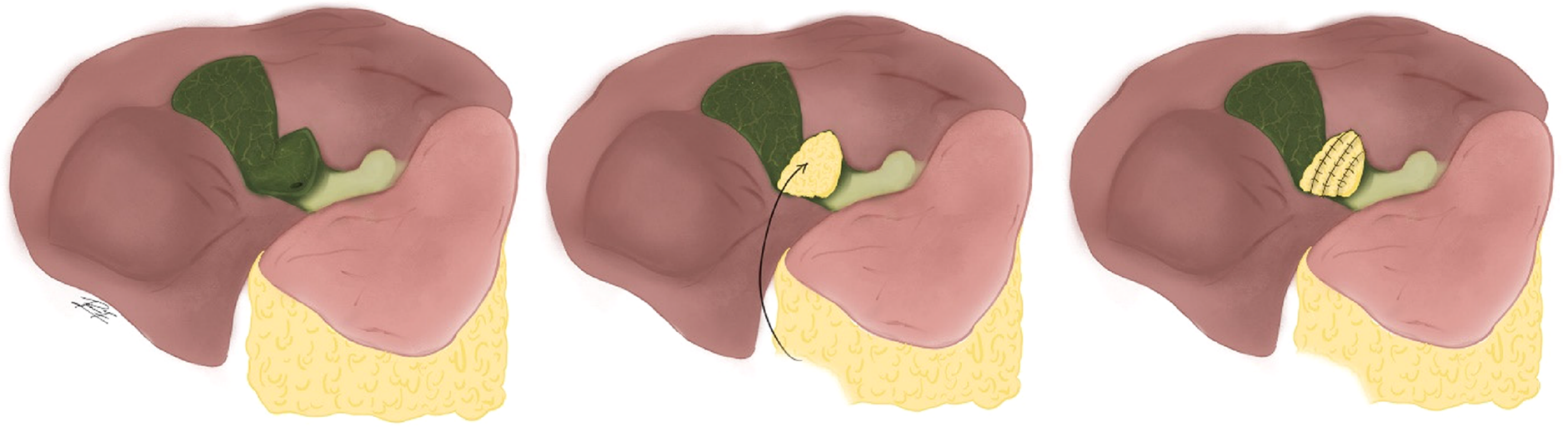
Free omental plugging.

Posterior to performing a fenestrating subtotal cholecystectomy, a piece of omentum that matches the approximate size of the gallbladder stump is resected from the greater omentum and plugged into the stump, oversewn with the edge of the stump and then both sides of the stump edges are closed as near to each other with absorbable sutures (Figure 6) (23).

**Figure 7:**
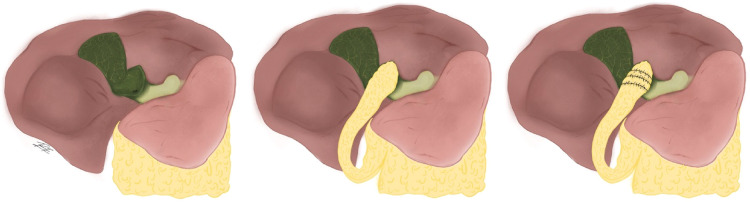
Pedicled omental flap.

With this technique bile leakage rates were of 6%, while bile leakage rates with no omental plugging were of 44% (23). This technique was performed in cases of fenestrating cholecystectomies, however it could still be performed in reconstituting cholecystectomies, because regardless of their lower rates of bile leakage it is still a possible complication. Non-vascularized, free omental tissue has been more accurately described however a vascularized omental pedicle can also be used (Figure 7) (24).

#### Robotic subtotal cholecystectomy

Robot assisted subtotal cholecystectomy has only been described in case reports. In one of the reported cases robot-assisted subtotal cholecystectomy was performed on a morbidly obese patient (body mass index of 59.9 m^2^/kg) where laparoscopic cholecystectomy may face multiple limitations. The patient was discharged on the first postoperative day and recovered without complications; however there still is not enough evidence to recommend or contraindicate its use (25).

### Controversies regarding the different surgical techniques

#### Outcomes of reconstituting and fenestrating subtotal cholecystectomy (Table 1)

Both subtotal cholecystectomy techniques were recently compared in a systematic review and meta-analysis which evidenced that fenestrating types were associated to higher rates of open conversion 10.2% vs. 4.2% (*p* < 0.001), retained stones 6.7% vs. 4.2% (*p* = 0.025), subhepatic or subphrenic collections 5.8% vs. 1.4% (*p* < 0.001), superficial surgical site infections 3.2% vs. 1.5% (*p* = 0.030), postoperative ERCP 14.4% vs. 6.6% (*p* < 0.001) and need for reoperation 3.5% vs. 1.3% (*p* < 0.001) in reconstituting types (26).

In another systematic review published in 2021 where both techniques were also compared fenestrating subtotal cholecystectomies were associated to bile leakage rates of 8.52% while reconstituting types had rates of 3.67%, being this the most frequent complication during subtotal cholecystectomies (8).

In a systematic review and meta-analysis by Nzenwa, et al., reconstituting cholecystectomies had a lower risk of bile leak, intraabdominal collection, intraabdominal infection, wound infection, nonsurgical wound infection, retained gallstones, recurrent biliary events, 30-day readmission, need for ERCP, percutaneous drainage and completion cholecystectomy (27).

During long-term follow-up (median follow-up time of 6 years), it was shown that episodes of recurrent biliary disease were less frequent with the fenestrating type compared to the reconstituting type (9% vs. 18%) (28).

It's important to consider that performing the reconstituting technique is not always possible due to gangrenous gallbladder walls, during which union is not possible thus not allowing proper closure of the gallbladder stump.

Modifications done to the fenestrating technique such as omental plugging and the falciform patch in order to avoid complications have not been performed in studies different to the ones described by the authors that have proposed them (Table 2).

**Table 2:** Summary of reconstituting vs. fenestrating subtotal cholecystectomy results.

Complications	Koo et al. (26)			Nzenwa et al. (27)		
	Reconstituting	Fenestrating	OR (CI95%)	Reconstituting	Fenestrating	RR (CI95%)
Bile leak	16.0% (150/935)	18.8% (107/570)	0.83 (0.63–1.09)	10.7% (291/2,719)	26.3% (214/815)	**0.41** **(0.34–0.49)**
Retained stones	4.1% (38/935)	6.7% (38/570)	**0.59** **(0.37–0.94)**	2.5% (68/2,719)	4.8% (39/815)	**0.52** **(0.33–0.81)**
Subhepatic or subphrenic collections	1.4% (13/935)	5.8% (33/570)	**0.23** **(0.12–0.44)**	1.9% (52/2,719)	3.6% (30/815)	**0.52** **(0.28–0.96)**
Wound infection	1.5% (14/935)	3.2% (18/570)	**0.47** **(0.23–0.94)**	2.6% (71/2,719)	5.5% (45/815)	**0.47** **(0.29–0.74)**
Need for reoperation	1.3% (12/935)	3.5% (20/570)	**0.36** **(0.17–0.74)**	NS	NS	NS
Need for ERCP	6.6% (62/935)	14.4% (82/570)	**0.42** **(0.30–0.60)**	3.7% (101/2,719)	15.2% (124/815)	**0.25** **(0.18–0.33)**
30-day mortality	0% (0/935)	0.7% (4/570)	0.07 (0.00–1.25)	NS	NS	NS

NS, not significant; bold values indicate statistically significant; (absolute values).

#### Should the gallbladder wall in contact with the liver be resected?

Resecting the gallbladder wall in contact with the liver has shown a lower risk of biloma, however, it increases the risk of intraabdominal infection, reintervention and recurrent biliary disease (27). However, dissecting the gallbladder wall in contact with the liver is not always feasible due to higher risk of hepatic bleeding in possible gallbladder intrahepatic presentation.

#### Should an open or laparoscopic approach be used?

Open subtotal cholecystectomy has been associated to higher risk of reintervention, surgical site infection and 30-day mortality, with lower risk of bile leakage compared to laparoscopic subtotal cholecystectomy (27, 29). Longer operation time, longer postoperative hospital stay and higher incisional hernia rates have also been reported in open subtotal cholecystectomy (29).

### Surgical outcomes

Subtotal cholecystectomy can sometimes be a challenging procedure; it's associated to a high rate of complications such as biliary fistula, retained gallstones, subhepatic or subphrenic collections, among others (22).

In a systematic review and meta-analysis by Elshaer et al., the main indications for performing this procedure were severe cholecystitis (72.1%), cholelithiasis in liver cirrhosis and portal hypertension (18.2%) and empyema or perforated gallbladder (6.1%). Morbidity rates for this procedure were low, some of them being: postoperative hemorrhage (0.3%), subhepatic collections (2.9%), bile duct injury (0.08%), retained stones (3.1%) while bile leakage rates were higher (18%). Reintervention was necessary in 1.8% of cases and mortality was 0.04%. When comparing an open vs. laparoscopic approach, laparoscopy had less risk of subhepatic collections, retained stones, wound infection, reoperation and mortality, although there was a higher risk of bile leakage (30).

In a recent systematic review and meta-analysis by Koo et al., open conversion rates were 7.7%, hemorrhage was 0.4%, bile duct injury was 0.3%, bile leak was 15.4%, retained stone was 4.6%, subhepatic or subphrenic collections were 2.9%, superficial surgical site infection was 2% and 30-day mortality was 0.2%. Additional procedures were sometimes needed such as ERCP in 8.8% of cases, percutaneous intervention in 1.1% and reoperation in 2.2% (26).

Another systematic review and meta-analysis by Nzenwa et al., that included 3,645 patients on which subtotal cholecystectomy was performed, bile duct injury was found in 0.2% of cases, vascular injury in 0.1%, bile leak in 13.9% and intraabdominal collections in 2.6%. Additional procedures were also documented such as ERCP in 6.9% of cases to treat bile leakage and retained stones, percutaneous drainage in 1.7% for collection drainage and reoperation in 1.0% be it either by laparoscopy or laparotomy. Documented mortality was of 0.3% (11/3,645) (27).

In a retrospective series which included 57 patients with an average follow-up time of 49 months, 5.3% presented symptomatic choledocholithiasis, 12.3% incisional hernia and 7% had symptomatic gallstones in the remnant tissue within the first year of surgery (31).

#### Bile leakage

One of many possible complications of cholecystectomy is bile leakage, and in most studies it was reported to be more frequent during subtotal cholecystectomy (10). Stent placement for bile leak is a treatment option with high success rates (32). However, ERCP is associated to additional costs, time and the possibility of complications such as pancreatitis (3.5%), bleeding (1.3%) and/or perforation (0.6%) (22, 33).

In most cases after performing subtotal cholecystectomy, especially in the fenestrating types, a drain is placed in the surgical site due to risk of bile leakage (34).

Bile secretion through the placed drain is expected when closing the cystic duct is not possible or when the procedure performed is a fenestrating type subtotal cholecystectomy, nonetheless it should gradually resolve by the 10th to 14th day (13). Due to this its preferrable to wait until bile secretion resolves on its own before considering ERCP with stent placement and/or sphincterotomy. Except for cases in which bile leakage is persistent, the patient lives in a remote location or if there are signs of infections or electrolyte disbalance, keeping the drain during the postoperative period is preferred over early ERCP (13).

Likewise as performing omental plugging or a falciform patch in order to reduce risk of bile leakage after subtotal cholecystectomy, some studies have attempted occlusion of the cystic duct with cyanoacrylate glue, yielding lower bile leak rates, hospital stay time and bile leak duration (35).

In a systematic review of the literature, the most frequent complication for subtotal cholecystectomy was biliary fistula, present in 12.2% out of 678 subtotal cholecystectomies evaluated. In this same study only 25.3% of these cases resolved spontaneously (8).

#### Retained gallstones in the remnant gallbladder

**Figure 8:**
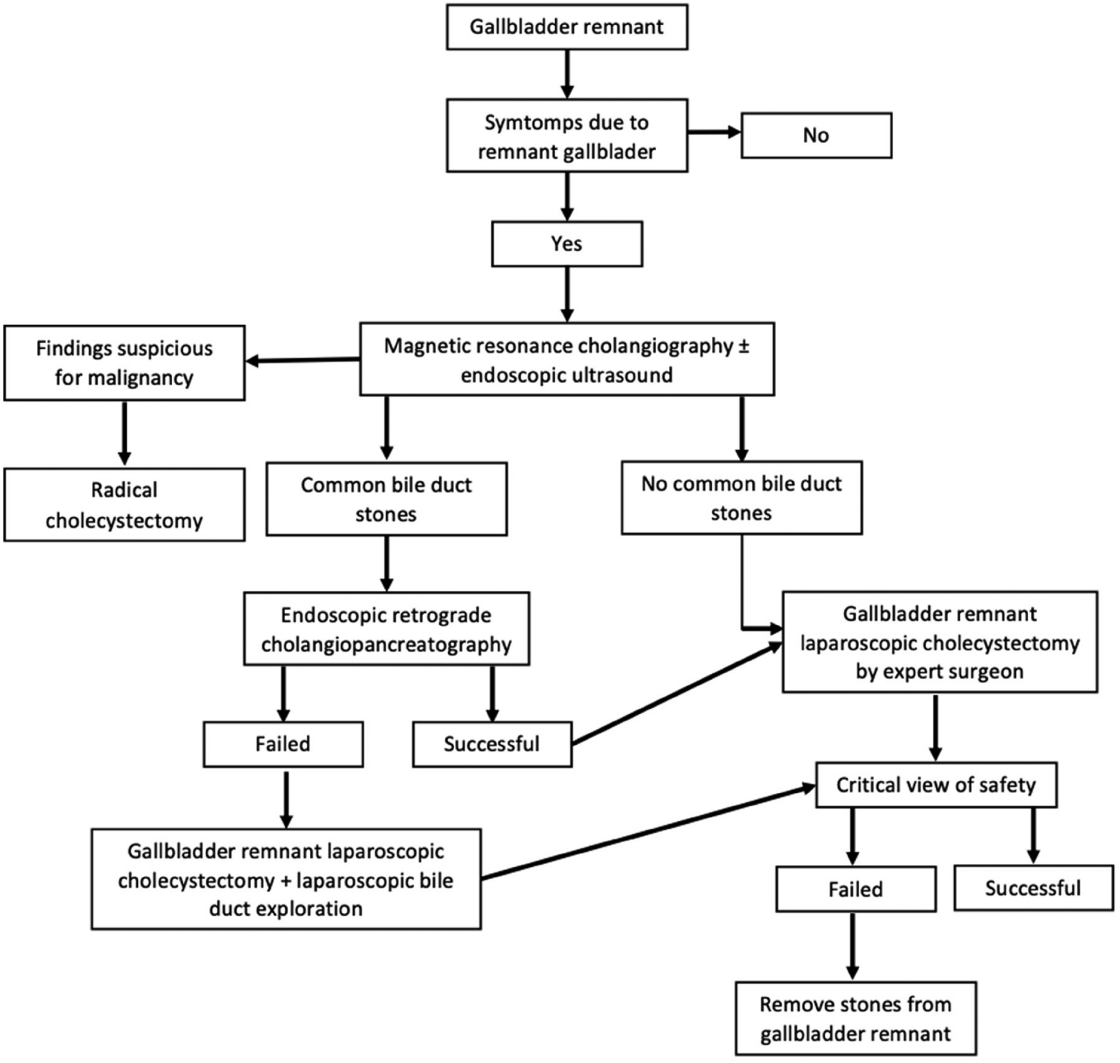
Treatment of recurrent gallstones.

Recurrent biliary disease associated to biliary dyskinesia, choledocholithiasis, retained gallstones and/or new stone formation in the gallbladder stump is a complication that must be considered after performing subtotal cholecystectomy. If symptoms are due to the presence of gallstones in the remnant gallbladder, complete cholecystectomy must be performed by a hepatobiliary or expert surgeon. Patients must also have a nuclear magnetic resonance before the procedure in order to be familiarized with the patient's anatomy. If reaching the critical view of safety is not possible during cholecystectomy of the remnant gallbladder it can be revised with mucosal cauterization and gallstone extraction while attempting to leave as small a remnant as possible (36). The following algorithm has been designed for treatment of recurrent gallstones (Figure 8) (36).

In the systematic review and meta-analysis by Nzenwa et al., retained gallstones in the remnant gallbladder or in the bile ducts were reported in 3.0% of patients, out of which 1.4% were symptomatic and 0.8% required extraction of the remnant gallbladder (27).

In a study that included 180 patients to which subtotal cholecystectomy was performed with a mean follow-up time of 880 days, there were retained gallstones in 29 patients, out of which 2 of them required a complete cholecystectomy of the remaining gallbladder (37). In another study that included 191 patients with a mean follow-up time of 2,195 days, recurrent biliary disease was reported in 12.6% of cases (28). In a populational study that included 210.719 cholecystectomies performed between 1998 and 2016, 0.25% required a second cholecystectomy (38).

In a case series which included 14 patients that required a second completion cholecystectomy after a previous subtotal cholecystectomy, indications for second cholecystectomy were cholecystitis in 5 cases, biliary cholic in 2 cases and biliary pancreatitis in 1 case. A laparoscopic approach was performed in 12 cases, out of which 5 needed conversion to open cholecystectomy, in the remaining 2 cases the procedure was performed with an open approach from the start. Results evidenced symptom relief in 13 out of 14 patients, morbidity was of 1 surgical wound infection and 1 bile duct injury (39). In another case series which included 11 patients taken to a second complete cholecystectomy, all cases had a gallbladder remnant of over 2.5 cm, which is considerably larger than recommended (<1 cm) (40).

#### Malignancy

One of the most infrequent complications that could happen after a subtotal cholecystectomy is the appearance of malignancy in the remaining portion of the gallbladder. Mucosal ablation of the stump using electrocautery should be considered as it may diminish presentation rates. However this has been rarely reported, possibly due to the lack of long-term follow-up (41).

## Discussion

Subtotal cholecystectomy proportions can reach up to approximately 10% (6–10) of all cholecystectomies, and as a result extensive knowledge on the subject by surgeons is imperative in order to yield the best results possible when performing this procedure.

It's important to evaluate the risk of facing a difficult cholecystectomy that may become a subtotal cholecystectomy before the procedure with the means of seeking support, stablishing an ideal schedule for the procedure, and informing family members. Multiple different scores that may help predict this risk have been designed because it's not dependent on one factor only, but rather on the sum of multiple ones and thus may be more determinative of difficult cholecystectomy (14, 42–45).

We consider that Lunevicius’ suggestion on a change of terminology regarding the method of subtotal cholecystectomy finalization, from the terms fenestrating and reconstituting subtotal cholecystectomies into open-tract and closed-tract cholecystectomy, respectively, as a more logical and intuitive naming of the procedure (2). The classification proposed by Purzner is the most complete and appropriate for reporting subtotal cholecystectomy (Figure 3) (20). Regarding the different possible variants on subtotal cholecystectomy resection, these can be correctly defined by using Lunevicius' classification (Figure 4) (21).

Whenever possible a subtotal closed-tract cholecystectomy should be performed considering that current literature reports a lesser rate of complications compared to subtotal open-tract cholecystectomy (26, 27). One of the most frequent complications is bile leak, so when subtotal closed-tract cholecystectomy is not possible an internal suture-closure of the cystic duct or a different technique such as an omental plugging or falciform ligament patch seem to be the best alternative for its prevention. However, available literature on both falciform ligament patch and omental plugging are limited and as a result more studies are needed to evaluate their effectivity. Another described alternative for bile leak prevention is the occlusion of the cystic duct using cyanoacrylate glue, however, this procedure isn't recommended because the glue can migrate to the bile duct and cause bile duct obstruction (22, 23, 35).

When performing subtotal closed-tract cholecystectomy using stapled sutures without opening the gallbladder and extracting the stones only, there remains a risk that the remaining gallbladder stump persist with gallstones due to an unsuccessful stone extraction. These remaining gallstones can be macerated by the stapled sutures producing micro stones that may migrate to the bile duct. As a result, the gallbladder should always be dissected in order to better inspect and extract all present stones. Additionally, the remaining gallbladder stump should be left as small as possible considering the risk of symptoms due to remnant gallbladder as these seem to be related to the size of the stump (40).

Subtotal cholecystectomy should be performed using a laparoscopic approach whenever possible; conversion to open procedure seems to be unnecessary and counterproductive because using this approach doesn't provide an adequate visualization of anatomical structures and the outcome for the procedure would still be a subtotal cholecystectomy. Furthermore, conversion to open procedure seems to be associated with higher rates of bile duct injury, bleeding, postoperative ileum, ICU admission and longer hospital stay (46).

Taking into account the high rates for bile leak after subtotal cholecystectomy regardless of its finalization technique (subtotal open-tract cholecystectomy and subtotal closed-tract cholecystectomy) (26, 27) we consider the use of a drain as the most effective choice for preventing bilioperitoneum. A comparison on conservative management using a drain vs. early ERCP should be evaluated in order to better define effectivity, complications and cost-efficiency.

When presenting persistent symptoms after cholecystectomy the patient's biliary anatomy should be evaluated using magnetic resonance cholangiography and endoscopic ultrasound, to discard choledocholithiasis and perform adequate presurgical planning for completion laparoscopic cholecystectomy of the gallbladder stump. When diagnostic image findings suggest malignant disease radical cholecystectomy in the hands of a hepatobiliary surgeon should be performed. The risk of malignancy after subtotal cholecystectomy should also be considered because mucosal ablation with electrocautery may diminish presentation rates. Studies evaluating long-term follow-up on the incidence and risk factors on malignancy of the gallbladder stump are needed considering its scarcity in current literature.

This study aimed to give an overview on subtotal cholecystectomy to inform on the different therapeutical options and outcomes available, as well as standardizing current nomenclature on the procedure with investigative purposes.

This review has some limitations. First, only English and Spanish language studies were included. Secondly the literature search may have missed some relevant studies. Thirdly, there was no formal evaluation of the quality of the included studies.

## Conclusion

Subtotal cholecystectomy is a safe alternative when facing difficult cholecystectomy in which the critical view of safety is not reached in order to avoid complications. A classification system should be implemented in surgical descriptions to compare the different surgical techniques employed. In order to avoid bile leakage and cholecystitis of the remnant gallbladder, the surgical technique must be performed skillfully. There is still a current lack of information on alternative techniques such as omental plugging or falciform patch in order to judge their utility. There needs to be further research on long-term complications such as malignancy of the remnant gallbladder.
